# Proteasome-Enriched hPPSCs-Derived EVs Attenuate Hypoxic Injury in Endothelial Cells via Proteasome-Mediated HIF-1α Degradation

**DOI:** 10.3390/ijms27157013

**Published:** 2026-08-04

**Authors:** Junyan Hao, Ying Wang, Youyu Ma, Shouting Liu, Yongsheng Gong, Junjun Xu

**Affiliations:** School of Basic Medical Sciences, Wenzhou Medical University, Wenzhou 325035, China; hjy729369@163.com (J.H.); w18331369530@163.com (Y.W.); mayouyu0225@163.com (Y.M.); liushouting@wmu.edu.cn (S.L.)

**Keywords:** extracellular vesicles, placental perivascular stem cells, proteomics, proteasome, endothelial cells, proteostasis

## Abstract

Stem cell-derived extracellular vesicles (EVs) hold therapeutic potential for hypoxia-associated injury, yet the molecular mechanisms underlying their protective effects remain incompletely defined. Herein, we performed comparative proteomic profiling of extracellular vesicles (EVs) secreted by human placenta-derived perivascular stem cells (hPPSCs) and human umbilical cord mesenchymal stem cells (hUCMSCs). We found that hPPSCs-EVs are enriched in proteasome-related proteins. Enzymatic activity assays further confirmed that proteasome activity in hPPSCs-EVs was significantly higher than that in hUCMSCs-EVs. Additionally, we observed that hPPSCs-EVs were efficiently endocytosed by human umbilical vein endothelial cells (hUVECs), leading to marked downregulation of hypoxia-inducible factor 1-alpha (HIF-1α) and intracellular ubiquitinated proteins. Importantly, this HIF-1α degradation persisted even when the host ubiquitin–proteasome system was blocked, indicating that hPPSCs-EVs can function independently of the host proteasomal pathway. These results reveal that hPPSCs-EVs deliver functional proteasomes to endothelial cells, thereby compensating for impaired protein degradation and protecting cells under hypoxic stress. Collectively, our findings provide evidence for an intercellular transfer of proteolytic capacity via stem cell-derived EVs, a mechanism that preserves endothelial proteostasis and highlights the therapeutic potential of proteasome-rich EVs for hypoxia-associated diseases.

## 1. Introduction

Extracellular vesicles (EVs) are small, membrane-bound structures that are released by cells into the extracellular milieu. EVs are categorized into exosomes, microvesicles, and apoptotic bodies according to their biogenesis pathways [1,2]. Exosomes are secreted upon the fusion between multivesicular bodies and the cytomembrane [3,4], whereas microvesicles and apoptotic bodies originate from direct outward sprouting [5,6]. EVs are characterized by a lipid bilayer membrane that encapsulates various biomolecules, encompassing nucleic acids, proteins, and lipids [7,8]. EVs play crucial roles in intercellular communication, immune modulation, and tissue development and are implicated in pathological conditions, such as cancer and angiocardiopathy [9].

The potential therapeutic role of EVs in hypoxic diseases has garnered considerable attention in recent years. EVs originating from oral and bone marrow mesenchymal stem cells have demonstrated protective benefits against hypoxia injury in cardiac myocytes [10,11]. Similarly, mesenchymal stromal cell-secreted EVs protect the brain from hypoxic–ischemic damage in neonatal mice and ovine fetuses [12,13], and mesenchymal stem cell-derived EVs attenuate hypoxia-induced apoptosis [14].

The ubiquitin–proteasome system (UPS) serves as the principal pathway for protein degradation in eukaryotic cells. The main component of the ubiquitin–proteasome system is the 26S proteasome, which comprises a catalytic 20S core and 19S regulatory complexes [15], with the 20S core being a conserved component [16]. The 20S proteasome complexes consist of four heteroheptameric rings, with the inner β-rings possessing various protease-like activities responsible for protein hydrolysis, while the α-rings regulate the entry of substrates [17]. The 19S proteasome consists of a base that facilitates substrate translocation, and a lid that recognizes substrates and removes ubiquitin [18]. As a key regulator of protein degradation and cellular homeostasis, proteasome dysfunction may lead to a spectrum of disorders, such as cardiovascular diseases, neurological disorders, and tumors.

Hypoxia-inducible factor 1 (HIF-1) is a pivotal molecular mediator in hypoxic response regulation. Typically, HIF-1 is a heterodimer consisting of the 1α and 1β subunits. Under normoxia, HIF-1α levels are controlled by prolyl hydroxylases (PHDs), which hydroxylate specific proline residues, facilitating recognition by von Hippel–Lindau (VHL) proteins, which induce the degradation of HIF-1α mediated by the ubiquitinated proteasome pathway [19,20]. During hypoxia, PHD activity diminishes, contributing to HIF-1α upregulation, which dimerizes with HIF-1β and activates genes involved in blood vessel formation, energy metabolism, and oxygen sensing [21,22]. Precise HIF-1α regulation is crucial for enabling cells to withstand damage induced by hypoxia and excessive reactive oxygen species (ROS), thereby maintaining cellular survival [23]. In comparison, aberrant HIF-1α levels worsen the severity of various pathological conditions [24]. For instance, high HIF-1α levels are detectable in the body fluids of patients with multiple inflammatory autoimmune diseases, such as systemic lupus erythematosus and rheumatoid arthritis [25]. Conversely, HIF-1α inhibition may mitigate the symptoms of such inflammatory diseases [26,27].

Currently, most research on proteasomes has focused on intracellular processes. However, some studies have indicated the existence of extracellular proteasomes, which also exert an effect on various physiological functions. Platelet-derived EVs contain proteasomes that participate in antigen presentation [28]. The therapeutic prospects of stem cell-derived EVs in hypoxic diseases has been extensively studied. However, studies exploring the use of EVs as carriers for the delivery of proteasomes to protect hypoxic cells are limited. Cellular hypoxia induces a series of stress responses that lead to significant alterations in endogenous proteasomes. Under acute oxidative stress, the 26S proteasome is reversibly degraded into the 20S proteasome and 19S complexes [29]. In addition, prolonged oxidative stress may impair 26S proteasome activity, leading to ubiquitinated protein aggregation [30]. Therefore, the use of EVs as carriers for exogenous proteasomes to regulate HIF-1α protein degradation in hypoxic cells may provide a novel therapeutic approach. CoCl_2_, as a hypoxia inducer, inhibits HIF-1α degradation, leading to its accumulation in cells [31,32]. In this study, we induced hypoxia in endothelial cells using CoCl_2_ and evaluated the impact of proteasomes delivered by human placenta-derived perivascular stem cells (hPPSCs)-EVs on HIF-1α degradation. In addition, we used MG-132, a reversible proteasome inhibitor that targets chymotrypsin-like activity [33,34], to assess the specific contribution of exogenous proteasomes delivered by hPPSCs-EVs. This study is the first to demonstrate that EVs encapsulating proteasomes are involved in HIF-1α degradation regulation in hypoxic endothelial cells. These findings have implications for treating ischemic and hypoxic disorders.

## 2. Results

### 2.1. Characterization and Proteomics of hPPSCs-EVs

**Figure 1:**
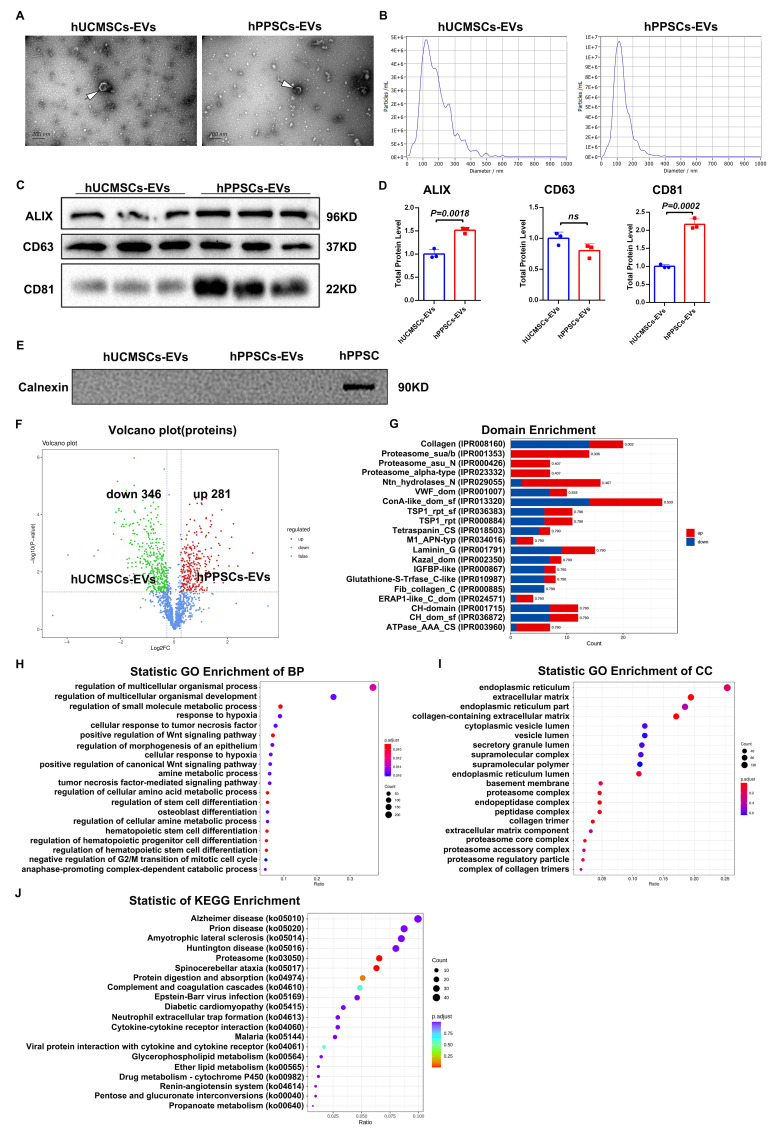
Characterization and proteomics of human placenta-derived perivascular stem cells (hPPSCs)–extracellular vesicles (EVs). (**A**) Transmission electron microscopy (TEM) characterization of EVs. Scale bar = 200 nm. The arrow is pointing at the EV. (**B**) Nanoparticle tracking analysis (NTA) of EVs. (**C**) WB analysis of ALIX, CD63, and CD81 in EVs. Equal total proteins were loaded per lane for EV samples from two stem cell types. (**D**) Quantitative analysis of ALIX, CD63, and CD81 protein levels. (**E**) WB analysis of Calnexin in EVs and hPPSCs. (**F**) Volcano plot analysis of differentially expressed proteins (DEPs) between hUCMSCs-EVs and hPPSCs-EVs. Green, red, and blue dots represent downregulated proteins, upregulated proteins, and proteins with no significant changes, respectively. The critical criteria were set to FC > 1.2 or FC < 0.83 (corresponding to |log_2_FC| > 0.263), with *p* < 0.05. (**G**) Protein domain analysis. (**H**,**I**) GO functional annotation biological process (BP) and GO functional annotation cell composition (CC) enrichment bubble analysis. The closer to red, the smaller the *p*-value. (**J**) KEGG enrichment analysis. The abscissa denotes the ratio, while the ordinate stands for the category. *n* = 3, *p* < 0.05.

hUCMSCs-EVs and hPPSCs-EVs were isolated by ultracentrifugation. Representative TEM micrographs showed that hPPSCs-EVs and hUCMSCs-EVs exhibited uniform cup shapes with distinct lipid bilayer membranes (Figure 1A). NTA results showed that for hPPSCs-EVs, the average particle diameter was measured as 131.2 nm, with a dominant peak at 112.1 nm accounting for 99.6% of the total particles, and the concentration was quantified as 1.20 × 10^11^ Particles/mL. In contrast, hUCMSCs-EVs exhibited a larger average diameter of 183 nm and a main peak at 123.5 nm (97.9% of total particles), with a lower concentration of 4.50 × 10^10^ particles/mL (Figure 1B). Western blotting confirmed the presence of the EV markers ALIX, CD63, and CD81. ALIX, an internal component involved in EV biogenesis [35], and the transmembrane proteins CD63 and CD81 were detected in both EV types (Figure 1C,D). Calnexin was not detected in either EV fraction, confirming the high purity of the preparations (Figure 1E). These results confirmed that the hPPSCs and hUCMSCs-secreted vesicles are extracellular vesicles, meeting the minimal characterization requirements recommended by the MISEV2023 guidelines [36], providing a reliable basis for subsequent experimental research. Proteins from hPPSCs-EVs and hUCMSCs-EVs were extracted and bioinformatically analyzed. Volcano plots displayed differentially expressed proteins (DEPs), alongside critical criteria were set to FC > 1.2 or FC < 0.83 (corresponding to |log_2_FC| > 0.263), with *p* < 0.05. A total of 627 proteins showed significantly differential expression, including 281 upregulated and 346 downregulated proteins. (Figure 1F) Top 20 proteins included collagen- and proteasome-related domains (e.g., proteasome_sua/b, proteasome_asu_N, and proteasome_alpha-type) (Figure 1G). Gene Ontology (GO) analysis revealed enrichment in the Extracellular Matrix (ECM), proteasome complex, and related proteasomal components, as well as in processes such as multicellular differentiation and small-molecule metabolism (Figure 1H,I). KEGG pathway analysis highlighted pathways such as Huntington’s, Alzheimer’s, prion disease, ALS, and proteasomes (Figure 1J). Overall, proteasome-related proteins were significantly enriched in the hPPSCs-EVs.

### 2.2. Active Proteasome Components Are Enriched in hPPSCs-EVs

**Figure 2:**
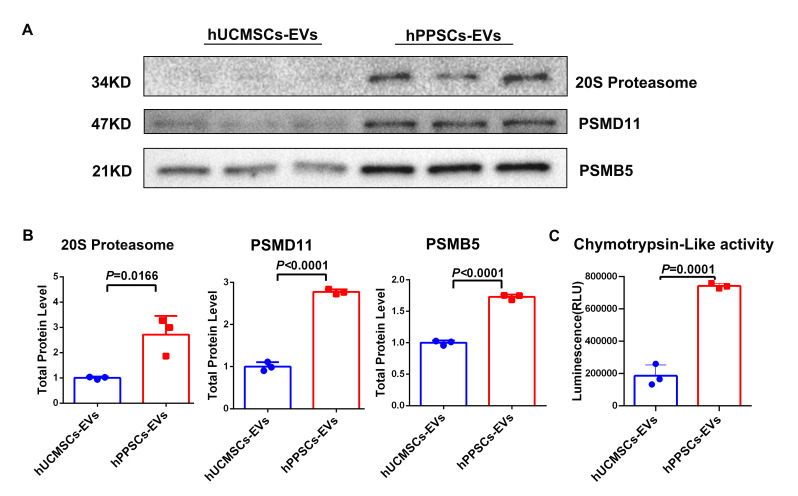
Proteasome composition detection and hPPSCs-EVs activity. (**A**) Protein expression levels of 20S proteasome, PSMD11, and PSMB5. (**B**) Quantitative analysis of 20S proteasome, PSMD11, and PSMB5 protein. (**C**) Proteasome chymotrypsin-like activity assay. *n* = 3, *p* < 0.05.

To further validate the proteomic findings, we assessed the levels of the 20S proteasome, β-subunit PSMB5, and PSMD11 subunit of the 19S proteasome in EVs. The results showed higher expression of the 20S proteasome (*p* = 0.0166), PSMD11 (*p* < 0.0001), and PSMB5 (*p* < 0.0001) in hPPSCs-EVs than those in hUCMSCs-EVs (Figure 2A,B). Additionally, the chymotrypsin-like proteasomal activity was significantly elevated in hPPSCs-EVs (*p* = 0.0001) (Figure 2C).

### 2.3. Proteasomes in hPPSCs-EVs Suppress HIF-1α Expression and CoCl_2_-Induced Ubiquitinated Protein Accumulation

**Figure 3:**
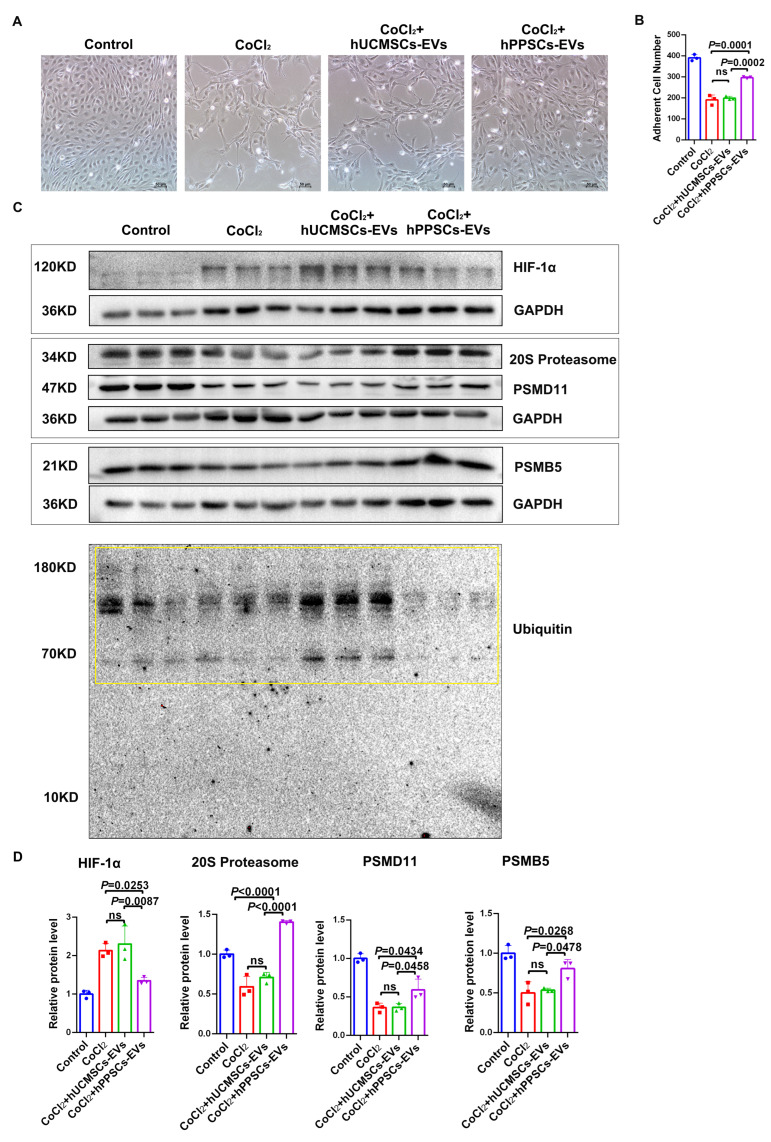
Proteasomes in hPPSCs-EVs suppress HIF-1α expression and ubiquitinated protein accumulation induced by CoCl_2_. (**A**) Representative micrographs of hUVECs in different treatment groups. Groups were set as follows: control hUVECs cultured in serum-free medium; CoCl_2_ hUVECs processed with 300 μM CoCl_2_ for 72 h; CoCl_2_+human umbilical cord mesenchymal stem cells (hUCMSCs)-EVs hUVECs treated with 300 μM CoCl_2_ and 75 μg/mL hUCMSCs-EVs for 72 h; and CoCl_2_+hPPSCs-EVs hUVECs treated with 300 μM CoCl_2_ and 75 μg/mL hPPSCs-EVs for 72 h (magnification, 200×; scale, 50 μm). (**B**) Quantitative analysis of the number of adherent endothelial cells, *n* = 3, *p* < 0.05. (**C**) The protein expression levels of HIF-1α, 20S proteasome, PSMD11, PSMB5, and ubiquitinated proteins. (**D**) Quantitative analysis of HIF-1α, 20S proteasome, PSMD11, and PSMB5 protein. *n* = 3, *p* < 0.05.

To evaluate the protective effects of hPPSCs-EVs under hypoxia, hUVECs were exposed to 300 μM CoCl_2_ (a hypoxia mimetic) with or without 75 μg/mL EVs for 72 h. Subsequently, cell morphology and adhesion were analyzed, and the expression of HIF-1α and proteasome-related proteins was assessed by Western blotting. Under a microscope, CoCl_2_-treated cells transformed from cobblestone-like to an elongated shape, and the number of adherent cells markedly decreased. In contrast, cells treated with hPPSCs-EVs exhibited a higher adherent cell count (Figure 3A,B). Western blot analysis revealed that HIF-1α was significantly downregulated in the hPPSCs-EVs group compared to those in the CoCl_2_ group (*p* = 0.0253) and hUCMSCs-EVs group (*p* = 0.0087). The hUCMSCs-EVs and CoCl_2_ groups did not differ significantly (Figure 3C,D). Additionally, expression levels of 20S proteasome, PSMD11, and PSMB5 were notably elevated in the hPPSCs-EVs group compared to those in the CoCl_2_ group (*p* < 0.0001, *p* = 0.0434, and *p* = 0.0268, respectively). Notably, the hPPSCs-EVs group also showed significant upregulation of these proteins compared to those in the hUCMSCs-EVs group (*p* < 0.0001, *p* = 0.0458, and *p*= 0.0478, respectively) (Figure 3C,D). Ubiquitinated protein accumulation decreased in the hPPSCs-EVs group compared to that in both the CoCl_2_ and hUCMSCs-EVs groups (Figure 3C).

### 2.4. Proteasomes in hPPSCs-EVs Inhibit MG-132-Induced HIF-1α Expression

**Figure 4:**
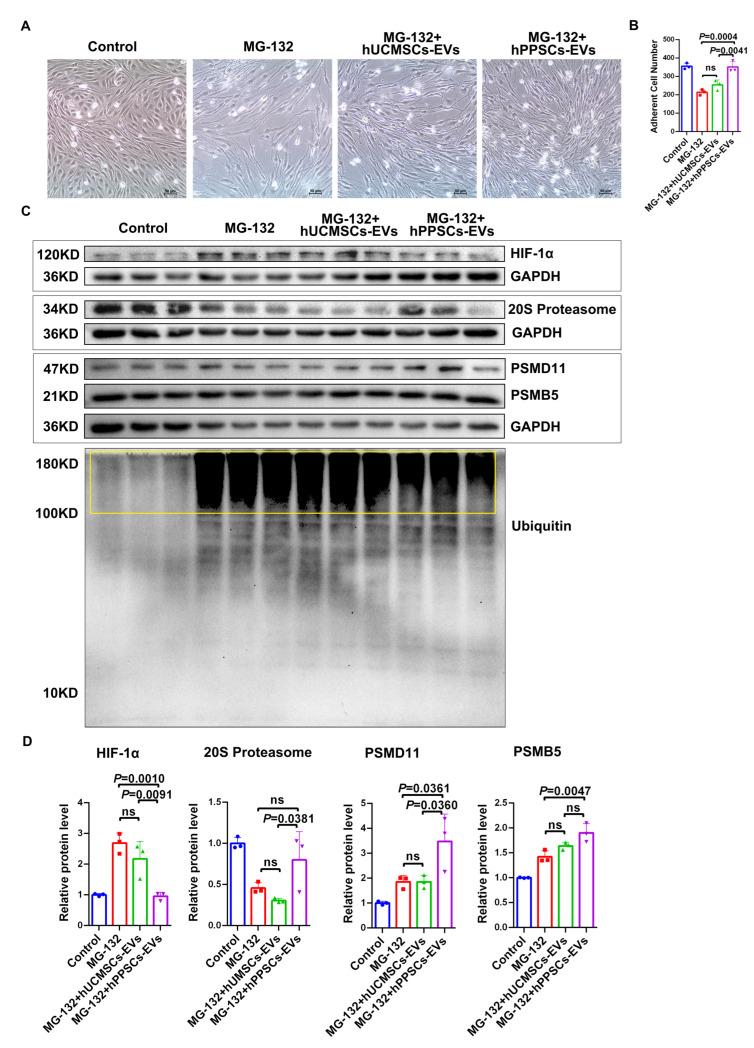
Proteasomes in hPPSCs-EVs inhibit MG-132-induced HIF-1α expression. (**A**) Representative micrographs of hUVECs in different treatment groups. Groups were set as follows: control hUVECs cultured in serum-free medium; MG-132 hUVECs subjected to 0.25 μM MG-132 for 72 h; MG-132+hUCMSCs-EVs hUVECs treated with 0.25 μM MG-132 and 75 μg/mL hUCMSCs-EVs for 72 h; and MG-132+hPPSCs-EVs hUVECs treated with 0.25 μM MG-132 and 75 μg/mL hPPSCs-EVs for 72 h (magnification, 200×; scale, 50 μm). (**B**) Quantitative analysis of the number of adherent endothelial cells, *n* = 3, *p* < 0.05. (**C**) The protein expression levels of HIF-1α, 20S proteasome, PSMD11, PSMB5, and ubiquitinated proteins. (**D**) Quantitative analysis of HIF-1α, 20S proteasome, PSMD11, and PSMB5 protein. *n* = 3, *p* < 0.05.

HIF-1α is hydroxylated by prolyl hydroxylase under normoxia, leading to ubiquitination via the VHL-E3 ligase complex and subsequent degradation by the proteasome [37]. To determine whether the proteasomes carried by hPPSCs-EVs function independently of the host ubiquitin–proteasome system, the endogenous degradation pathway was blocked using 0.25 μM MG-132 under normoxic conditions for 72 h, with or without 75 μg/mL EVs. Microscopy revealed that MG-132 treatment caused cell sparsity and morphological changes from cobblestone to elongated shapes, along with a marked reduction in adherent cell counts. However, endothelial cells incubated with hPPSCs-EVs showed a significant increase in adherent cell counts compared to MG-132 and hUCMSCs-EV treatments. (Figure 4A,B).

Western blot analysis showed no significant difference in HIF-1α levels between the hUCMSCs-EVs and MG-132 groups. However, HIF-1α was notably downregulated in the hPPSCs-EVs group compared to those in both the MG-132 (*p* = 0.0010) and hUCMSCs-EVs groups (*p* = 0.0091) (Figure 4C,D).

Further analysis demonstrated that the expression levels of PSMD11 (*p* = 0.0361) and PSMB5 (*p* = 0.0047) were significantly higher in the hPPSCs-EVs group than in the MG-132 group (Figure 4C,D). No significant differences were noted between the hUCMSCs-EVs and MG-132 groups. Additionally, the hPPSCs-EVs group showed increased levels of the 20S proteasome (*p* = 0.0381) and PSMD11 (*p* = 0.0360), but not PSMB5, compared to that in the hUCMSCs-EVs group (Figure 4C,D). Less ubiquitinated proteins (100–180 kDa) accumulated in the hPPSCs-EVs group than in both the MG-132 and hUCMSCs-EVs groups (Figure 4C). Collectively, these findings support that proteasomes in hPPSCs-EVs mediate the degradation of HIF-1α under normoxic conditions.

### 2.5. hPPSCs-EVs Proteasomes Degrade HIF-1α Accumulated Under Both CoCl_2_ and MG-132

**Figure 5:**
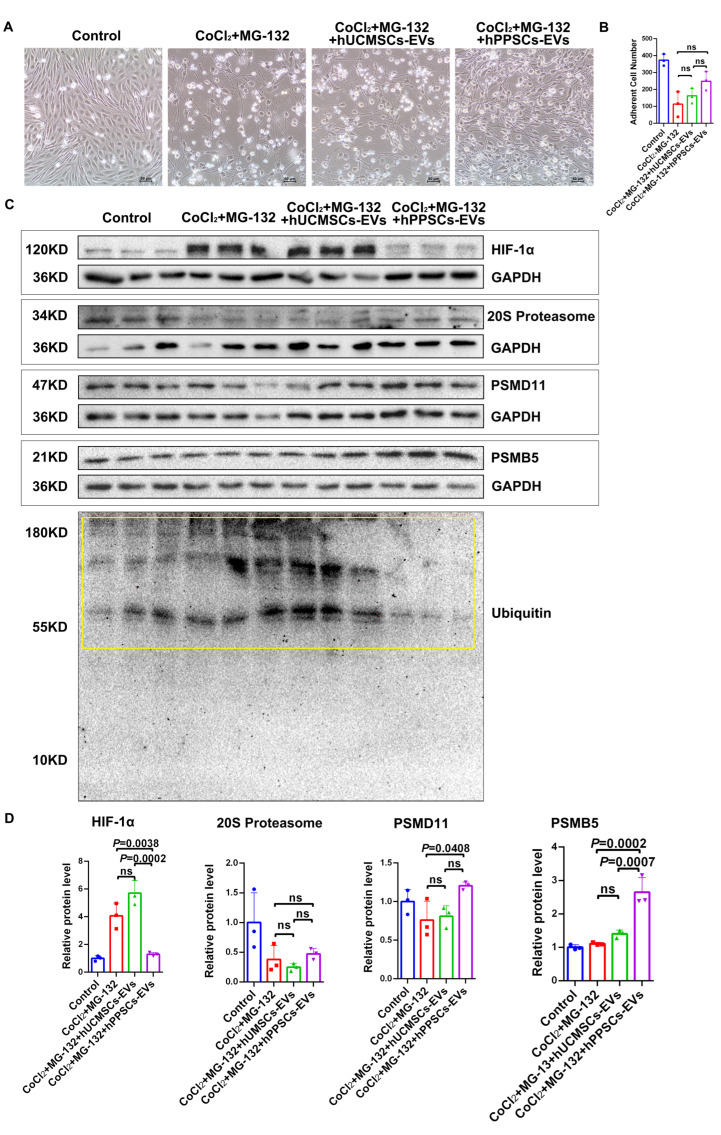
The hPPSCs-EVs proteasomes degrade HIF-1α induced by both CoCl_2_ and MG-132. (**A**) Representative micrographs of hUVECs in different treatment groups. Groups were set as follows: control hUVECs cultured in serum-free medium; CoCl_2_+MG-132 hUVECs treated with 300 μM CoCl_2_ and 0.25 μM MG-132 for 72 h; CoCl_2_+MG-132+hUCMSCs-EVs hUVECs treated with 300 μM CoCl_2_, 0.25 μM MG-132, and 75 μg/mL hUCMSCs-EVs for 72 h; and CoCl_2_+MG-132+hPPSCs-EVs hUVECs treated with 300 μM CoCl_2_, 0.25 μM MG-132, and 75 μg/mL hPPSCs-EVs for 72 h. (magnification: 200×; scale bar: 50 μm) (**B**) Adherent endothelial cell count quantitation, *n* = 3, *p* < 0.05. (**C**) The protein expression levels of HIF-1α, 20S proteasome, PSMD11, PSMB5, and ubiquitinated proteins. (**D**) Quantitative analysis of HIF-1α, 20S proteasome, PSMD11, and PSMB5 protein. *n* = 3, *p* < 0.05.

To determine whether hPPSCs-EVs preserve endothelial cell homeostasis under the combined stress of hypoxia and proteasome blockade, hUVECs were co-treated with CoCl_2_ (300 μM) and MG-132 (0.25 μM) for 72 h, in the presence or absence of 75 μg/mL EVs. Compared with the control group, exposure to CoCl_2_ combined with MG-132 resulted in a significant reduction in adherent cell numbers. Treatment with hUCMSCs-EVs failed to significantly attenuate this injury. Notably, although the differences between the hPPSCs-EVs-treated group and either the CoCl_2_+MG-132 group or the hUCMSCs-EVs group did not reach statistical significance, hPPSCs-EVs treatment still exhibited a trend toward increased cell adhesion (Figure 5A,B). Western blot analysis revealed no notable disparity in HIF-1α levels between the hUCMSCs-EVs and MG-132+CoCl_2_ groups. However, HIF-1α was significantly downregulated in the hPPSCs-EVs group compared to both the MG-132+CoCl_2_ (*p* = 0.0038) and hUCMSCs-EVs groups (*p* = 0.0002) (Figure 5C,D).

Additionally, expression of the 20S proteasome, PSMD11, PSMB5, and ubiquitinated proteins was assessed. PSMD11 (*p* = 0.0408) and PSMB5 (*p* = 0.0002) were markedly elevated in the hPPSCs-EVs group compared to those in the MG-132+CoCl_2_ group (Figure 5C,D). No significant differences were observed between the hUCMSCs-EVs and the MG-132+CoCl_2_ groups. Moreover, PSMB5 expression was significantly higher in the hPPSCs-EVs group compared to the hUCMSCs-EVs group (*p* = 0.0007), whereas PSMD11 showed no difference (Figure 5C,D). Ubiquitinated protein accumulation was reduced in the hPPSCs-EVs group relative to the other groups (Figure 5C). Overall, these findings highlight the efficacy of hPPSCs-EVs in facilitating HIF-1α degradation under both hypoxic and proteasomal inhibition conditions, emphasizing their unique capacity to modulate cellular protein homeostasis in response to dual stressors.

## 3. Discussion

The mechanisms by which extracellular vesicles from stem cells exert their effects in hypoxic diseases have been partially elucidated. However, the potential of EVs to protect cells through proteasome transport, particularly endothelial cells under hypoxic conditions, remains unclear. As the vascular barrier’s core components, endothelial cells are critical for tissue perfusion and damage repair, and their survival under hypoxia directly influences these processes. Through the degradation of aberrant proteins, the proteasome maintains cellular homeostasis and may represent a key target through which EVs exert their protective effects. This study focused on whether hPPSCs-EVs could modulate the survival of endothelial cells under hypoxic conditions via proteasome transport, with the aim of providing a novel mechanistic perspective on the role of EVs in hypoxic injury.

This study characterized hPPSCs-EVs and identified high ALIX and CD81 expression levels. (Due to the absence of commonly present housekeeping proteins in EV samples [38], an equal amount of total protein was loaded to ensure equal loading between the two EV types.) As important components of EVs, CD63 and CD81 are transmembrane proteins of EVs, whereas ALIX, as an internal component of EVs, is involved in EV biogenesis [39,40,41]. ALIX is a cytoplasmic protein that targets late endosomes [42] and may drive intraluminal vesicle (ILV) formation in multivesicular bodies (MVBs) by directly binding to ESCRT-III subunit CHMP4 or by aggregating ubiquitinated MVB membrane proteins [43]. These findings suggested that hPPSCs have a strong ability to secrete EVs.

After characterizing hPPSCs-EVs, proteomic analysis revealed the enrichment of proteasome-related proteins. These findings suggest that hPPSCs-derived EVs serve as key contributors to tissue repair, maintenance of cellular microenvironment homeostasis, regulating protein degradation, and post-translational modifications. Western blotting and activity assays confirmed the high expression of active 20S proteasomes in hPPSCs-EVs, and detection of the 19S proteasome subunit PSMD11 indicated the presence of 19S particles or even intact 26S proteasomes. Collectively, these results further indicate that hPPSCs-EVs possess an enhanced proteasome machinery and that active protein degradation pathways exist within the EVs. Proteasomes regulate cellular protein homeostasis by selectively degrading the ubiquitinated proteins [44]. The 20S proteasome can independently degrade various proteins without relying on 19S regulatory particles, ubiquitination, or ATP [45]. Furthermore, the free 20S proteasome exhibits high structural stability and enzymatic activity in the extracellular environment [46]. Given the spatial constraints of the EV, the molecular weight of the 20S proteasome is significantly lower than that of the 26S proteasome [45,47]. Therefore, we hypothesized that the proteasome in hPPSCs-EVs is primarily the 20S proteasome.

Based on these observations, we focused on the role of hPPSCs-EVs-derived proteasomes in HIF-1α degradation. Under normoxic, HIF-1α is degraded by proteasomes [48]. In contrast, inhibition of PHDs under hypoxia leads to HIF-1α accumulation [23], a process mimicked by CoCl_2_, which suppresses HIF-1α degradation [49,50]. Herein, hUVECs were treated with CoCl_2_ to induce hypoxia, with 75 µg/mL of hPPSCs-EVs or hUCMSCs-EVs added simultaneously. This concentration was selected based on previous studies, which showed that mesenchymal stem cell-derived EVs (MSC-EVs) in the range of 50–100 µg/mL exhibited strong biological activity, including anti-inflammatory, tissue repair, and pro-regenerative effects [51,52,53]. Therefore, the intermediate concentration of 75 µg/mL throughout this research was selected to ensure the reliability and scientificity of the experimental design. HUVECs cultured in serum-free ECM medium served as the blank control group in this study, and all other treatment groups were also uniformly maintained in serum-free culture systems for subsequent interventions. This experimental design can eliminate the interference of serum-derived extracellular vesicles and is consistent with existing research on the regulation of endothelial cells by mesenchymal stem cell-derived extracellular vesicles [54,55,56], thereby enhancing the specificity and reliability of the experimental results. The results showed that HIF-1α expression was upregulated, whereas HIF-1α level was decreased after the treatment with hPPSCs-EVs. This suggests that hPPSCs-EVs can inhibit HIF-1α expression. However, the specific cargo molecules responsible for this effect remain unidentified. By integrating proteomic data, we propose that this effect is mediated by hPPSCs-EVs-derived proteasomes. Subsequently, cells treated with hPPSCs-EVs after CoCl_2_ exposure showed high expression of the 20S proteasome, PSMB5, and the subunit of 19S-regulated granules, PSMD11, and decreased accumulation of ubiquitinated proteins. This demonstrates that hPPSCs-EVs deliver exogenous proteasomes to target cells, degrade intracellular proteins, and compensate for the impaired proteasome function in hypoxia-damaged cells.

To further verify the function of these proteasomes, we used the proteasome inhibitor, MG-132. Under normoxic circumstances, the MG-132 treatment group exhibited significant PSMB5 downregulation, indicating effective proteasome inhibition within endothelial cells. Concurrently, elevated levels of HIF-1α and ubiquitinated proteins were observed, suggesting a blockade of the ubiquitination degradation pathway of proteins. In contrast, treatment with hPPSCs-EVs upregulated 20S proteasome, PSMB5, and PSMD11, with reduced HIF-1α and ubiquitinated proteins. These findings demonstrate that hPPSCs-EVs replenish proteasomes and restore their degradation function, and highlight the compensatory role of EV-derived proteasomes in intracellular system failure.

Considering the limitations of individual treatments, this study used a combination treatment with CoCl_2_ and MG-132 to comprehensively verify the function of proteasomes in hPPSCs-EVs. This approach aims to eliminate interference from endogenous proteasomes and single environmental factors, allowing for a direct and unambiguous verification of their function in complex scenarios. This provides a reliable experimental basis for fully validating the delivery of exogenous proteasomes by hPPSCs-EVs to degrade excessively accumulated proteins and provide a protective effect. These results indicated that proteasomes derived from hPPSCs-EVs retained their degradation capability. However, due to the inhibition of PHD by CoCl_2_, the mechanism of downregulation of HIF-1α by the proteasome derived from hPPSCs-EVs requires additional investigation. Currently, two pathways, CHIP-Hsp70 [57,58] or RACK1-Hsp90 [59], have been identified, which are O2/PHD/VHL pathway-independent for HIF-1α degradation [60]. Therefore, we hypothesize that hPPSCs-EVs-derived proteasomes may degrade HIF-1α through these two alternative pathways. Notably, in this study, we compared hPPSCs-EVs with hUCMSCs-EVs and found that hPPSCs-EVs exhibited higher expression levels of the 20S proteasome and PSMB5 along with enhanced proteasomal activity. Additionally, hPPSCs-EVs significantly downregulated HIF-1α and ubiquitinated protein expression compared to those in hUCMSCs-EVs.

hUCMSCs were selected as the comparator cell type in this study because they possess certain therapeutic potential and are widely available in regenerative medicine, although they also face some challenges [61]. Notably, our comparative proteomic analysis revealed that hPPSCs-EVs are intrinsically enriched in proteasome-related proteins compared with hUCMSCs-EVs. This molecular difference likely accounts for the superior protective efficacy of hPPSCs-EVs observed in the functional assays. It is well known that the protein composition of different EVs is not entirely identical, and that the biological functions of EVs are largely determined by their protein cargo [62]. In this context, the robust proteasome activity delivered by hPPSCs-EVs, as confirmed by enzymatic activity assays, may efficiently compensate for the impaired host ubiquitin–proteasome system under hypoxic stress, thereby promoting targeted degradation of HIF-1α and maintaining endothelial homeostasis. In contrast, the relatively low abundance of proteasome components in hUCMSCs-EVs limits their capacity to mitigate hypoxia-induced injury.

Why do hPPSCs-EVs exhibit a more pronounced proteasome-mediated effect compared with hUCMSCs-EVs? This discrepancy may be closely related to their distinct tissue origins and corresponding biological properties. The placenta is intrinsically specialized in maintaining vascular homeostasis and functions as a highly active secretory organ throughout pregnancy, continuously releasing diverse bioactive molecules into the maternal circulation. This unique physiological role may naturally endow hPPSCs-EVs with a richer cargo of proteasome-related proteins and other tissue-repair factors. In contrast, hUCMSCs are derived from the umbilical cord, a tissue more closely associated with immunomodulatory functions and less specialized in vascular maintenance. This functional divergence between the parental cells likely accounts for the comparatively weaker proteasome activity and protective efficacy observed in hUCMSCs-EVs. Further mechanistic analysis reveals that, as blood-borne vascular pericytes, the primary physiological task of hPPSCs is to maintain vascular homeostasis—regulating blood flow, preserving barrier integrity, and participating in angiogenesis [63,64]. The EVs they secrete may require a higher proteasome content to rapidly degrade misfolded proteins, thereby ensuring stable function. In contrast, although MSC-EVs do carry proteasomes [65], their biological functions are more focused on remote tissue repair and immunomodulation through cargos such as miRNAs and growth factors [66,67], with a relatively lower demand for the immediate degradation of harmful components. This functional divergence may directly influence the basal set point of proteasomal activity in these two cell types. As hPPSCs are chronically adapted to the demands of rapid protein turnover and quality control within the circulatory microenvironment, they appear to maintain a higher basal proteasomal activity. Collectively, it is tempting to speculate that the more pronounced proteasomal activity observed in hPPSCs may represent, at least in part, an adaptive consequence of their long-term exposure to the blood-borne circulatory microenvironment. Additionally, differences in EV uptake efficiency or uptake mechanisms between the two EV types cannot be excluded and warrant further investigation [68]. We acknowledge this as a limitation of the present study. Accordingly, we plan to perform flow cytometry-based uptake assays in future work to obtain more rigorous quantitative data and further validate the internalization efficiency of hPPSC-EVs.

## 4. Materials and Methods

### 4.1. Cell Culture and Experimental Treatment

Human umbilical vein endothelial cells (HUVECs) were cultured in Endothelial Cell Medium (ECM) (ScienCell, Cat. 1001, Carlsbad, CA, USA) within sterile Petri dishes (Jet Biofil, TCD000100, Guangzhou, China) at a density of 1.3 × 10^6^ cells per dish, under 5% CO_2_ at 37 °C. The medium was changed every 2 days, and cells were passaged every 3–4 days. Upon reaching the third passage, cells were subcultured into six-well plates (Nest, Cat. 701001, Woodbridge, NJ, USA) at a density of 1 × 10^5^ cells per well when 70–80% confluent. Cells were subsequently stimulated with 300 μM CoCl_2_ (Macklin, C885204, Shanghai, China) to induce hypoxia or with 0.25 μM MG-132 (MCE, HY-13259, Monmouth Junction, NJ, USA) to inhibit proteasomes for HIF-1α degradation. Simultaneously, cells were treated with either human umbilical cord mesenchymal stem cells (hUCMSCs)–EVs or hPPSCs-EVs at 75 μg/mL for 72 h to evaluate the role of the hPPSCs-EVs-derived proteasome in HIF-1α expression. HUVECs cultured in serum-free ECM medium served as the blank control group in this study, and all other treatment groups were also uniformly maintained in serum-free culture systems for subsequent interventions. The experiments were performed in triplicates.

### 4.2. EVs Separation and Concentration

hPPSCs and hUCMSCs were cultured as previously described [69], which was as followed: Placenta was collected in a sterile bag and transported to the lab at 4 °C. After washing with PBS containing 2% Pen/Strep to remove blood, terminal villi rich in capillaries were dissected using sterile scissors and forceps. Villi were digested with 1 mg/mL collagenase II and IV at 37 °C for 60 min, with gentle mixing every 10 min. Digestion was stopped by adding DMEM/F12 with 10% FBS. After adding equal PBS, the mixture was sedimented for 5 min; the pellet was discarded. This sedimentation was repeated twice. The supernatant was then filtered through 100, 70, and 40 µm strainers. Microvascular fragments (40–70 µm) were collected, resuspended in pericyte medium with PGS, and cultured in Petri dishes at 37.5 °C with 5% CO_2_. Medium was changed every 2 days, and cells were passaged every 3–4 days. The complete PM medium was formulated with 500 mL basal medium, 10 mL fetal bovine serum (FBS, Cat. No. 0010), 5 mL pericyte growth supplement (PGS, Cat. No. 1252), and 5 mL penicillin/streptomycin solution (P/S, Cat. No. 0503). Upon reaching passage 3 (P3), the cells were switched to serum-free medium (without additional serum supplementation) and cultured continuously, after which the extracellular vesicles were subsequently collected. hUCMSCs’ culture: The umbilical cord tissue had arteries and veins removed, Wharton’s jelly was isolated, and the tissue was minced. The tissue blocks were incubated in MSC medium (MSCM, ScienCell, Cat. 7501). MSCM consists of 500 mL basal medium, 25 mL fetal bovine serum (FBS, Cat. No. 0025), 5 mL mesenchymal stem cell growth supplement (MSCGS, Cat. No. 7552), and 5 mL penicillin/streptomycin solution (P/S, Cat. No. 0503). And once the blocks adhered to the culture surface, medium was replaced every 2 days. Similarly, upon reaching passage 3 (P3), the cells were switched to serum-free medium (without additional serum supplementation) and cultured continuously, after which the extracellular vesicles were subsequently collected. The collected medium was used for EVs isolation via differential ultracentrifugation. Briefly, the media were centrifuged at 4 °C, 10 min at 3000× *g*, followed by 30 min at 12,000× *g*. The supernatants were decanted into 40 mL centrifuge tubes (Beckman, 355654, Brea, CA, USA) and ultracentrifuged at 150,000× *g* for 70 min at 4 °C using an ultracentrifugation centrifuge equipped with a Type 70 Ti rotor (Beckman, Beckman Otima100, Brea, CA, USA). The resulting EV pellets were resuspended in PBS, mixed thoroughly, and stored in 1.5 mL sterile tubes at −80 °C for subsequent analyses.

### 4.3. Transmission Electron Microscopy (TEM)

In total, 10 µL of the extracellular vesicle solution was dispensed onto a copper mesh and incubated for 10 min at room temperature. Subsequently, 10 µL of 2% uranyl acetate was applied onto the mesh for 1 min of negative staining. Residual staining solution was blotted off with filter paper, followed by 2 min air-drying under an incandescent lamp. Finally, the mesh was mounted onto the transmission electron microscope (H-7650, Hitachi High-Tech Corporation, Tokyo, Japan) and imaged at 80 kV accelerating voltage.

### 4.4. Nanoparticle Tracking Analysis (NTA)

Extracellular vesicle concentration, size distribution, and particle motion trajectories were determined using ZetaView^®^ Nanoparticle Tracking Analyzers (S/N 18-373, Particle Metrix, Inning am Ammersee, Germany). Measurements were conducted at 25.11 °C. Specifically, the EV suspension was illuminated with a laser light source, and the light scattered by the nanoparticles was detected. The concentration of EV was determined by counting the number of scattered particles. The EV suspension was diluted with PBS to a concentration of 1 × 10^7^–1 × 10^9^ particles/mL, and its particle motion trajectories and size were analyzed. The specific parameters were as follows: laser wavelength of 488 nm; ZetaView software version 8.04.02 SP2; camera settings of frame rate of 30 fps, gain of 19.2, offset of 0, and shutter of 70; analysis settings of minimum brightness of 20, minimum area of 5, maximum area of 1000, maximum brightness of 255, and minimum trace length of 15; and an average of approximately 203 particles per frame.

### 4.5. Western Blot

EVs and cells were homogenized using radio immunoprecipitation assay (RIPA) buffer (Beyotime, P1048, Shanghai, China) and iced for 5 min, followed by centrifugation at 13,000 rpm for 15 min (4 °C) to collect the supernatant. Protein concentrations were quantified via bicinchoninic acid (BCA) assay kit (ThermoScientific, 23225, Cambridge UK). Equal protein aliquots were loaded onto SDS-PAGE gels, separated, and then transferred onto polyvinylidene fluoride (PVDF) membranes (Immobilon, ISEQ00010, Merck KGaA, Darmstadt, Germany). After blocking with 5% skim milk (Beyotime, P0216) for 1 h at room temperature and then rinsed thrice with Tris-buffered saline+Tween-20 (TBST). Overnight incubation of primary antibodies was conducted at 4 °C, followed by washes with TBST (four times). Membranes were then incubated with HRP-conjugated secondary antibodies at room temperature for 1 h. All protein bands were visualized via NcmECLUltra reagent (NCM Biotech, P10100, Suzhou, China) and a multi-function gel imaging system (BIO-RAD, ChemiDoc, Hercules, CA, USA), and band intensities were quantified via ImageJ (NIH, version 1.53c). GAPDH was employed as the internal control. The antibodies employed for Western blot in this study are listed below: Anti-ALIX antibody (Abcam, ab275377, rabbit, Cambridge, MA, USA), diluted at 1:1000; Anti-CD81 antibody (ABclonal, A5270, rabbit, Woburn, MA, USA), diluted at 1:1000; Anti-CD63 antibody (ABclonal, A19023, rabbit), diluted at 1:1000; Anti-HIF-1α antibody (ABclonal, A16873, rabbit), diluted at 1:1000; Anti-Ubiquitin (P4D1) antibody (SANTA, sc-8017, mouse), diluted at 1:1000; Anti-PSMD11 antibody (ABclonal, A15306, rabbit), diluted at 1:500; Anti-PSMB5 antibody (ABclonal, A1975, rabbit), diluted at 1:1000; Anti-Proteasome 20S alpha + beta antibody (Abcam, ab22673, rabbit), diluted at 1:1000; Anti-GAPDH antibody (ABclonal, A19056, rabbit), diluted at 1:5000; Anti-Calnexin antibody (proteintech, 10427-2-AP, rabbit), diluted at 1:10,000; HRP-linked anti-rabbit IgG (CST, 7074, goat), diluted at 1:10,000; and HRP-linked anti-mouse IgG (CST, 7076, horse), diluted at 1:10,000. Prior to incubation with the PVDF membrane, all primary and secondary antibodies were diluted in TBST buffer.

### 4.6. Proteomics

#### 4.6.1. Sample Preparation

Proteomic analysis was conducted by Meteville Biotechnology Company (Wuhan, China). The hUCMSCs-EVs and hPPSCs-EVs were combined with 8M urea buffer containing 100 mM Tris-Cl and processed via water bath ultrasonication, followed by incubation with dithiothreitol (DTT) for 1 h at 37 °C. Next, iodoacetamide (IAA) was introduced to perform alkylation, and the Bradford method was applied to assay protein concentrations of all samples. A 100 mM Tris HCl solution was then supplemented to dilute the urea concentration to less than 2 M, and trypsin was incorporated at an enzyme-to-protein mass ratio of 1:50, followed by continuous incubation at 37 °C overnight. Trifluoroacetic acid (TFA) was infused the next day to terminate digestion. The obtained supernatant underwent Sep-Pak C18 desalting treatment and purification, vacuum-dried, and then stored at −20 °C. Equal amounts of each sample were subjected to TMT labeling. Following re-desalting, the samples were fractionated using high-pH reverse-phase chromatography. Each individual fraction was vacuum-dried again and preserved at −80 °C until mass spectrometry (MS) detection.

#### 4.6.2. LC-MS/MS Assay

Data were acquired using a liquid chromatography–tandem mass spectrometry (LC-MS/MS) system comprising a Q Exactive Plus mass spectrometer (Thermo Fisher Scientific (Bremen) GmbH, Bremen, Germany) integrated with an EASY-nLC 1200 liquid chromatograph (Thermo Fisher Scientific, Waltham, MA, USA). The peptide samples were loaded onto a 50 μm × 15 cm C18 analytical column (2 μm, 100 Å) for fractionation. Two mobile phases were utilized to build the separation gradient: Phase A comprised 0.1% formic acid, and Phase B was prepared with 80% ACN plus 0.1% formic acid, with flow rate adjusted to 300 nL/min. Under data-dependent acquisition (DDA) mode, each scanning cycle comprises a single full-scan mass spectrum (resolution, 70,000; AGC target, 3 × 10^6^; max IT, 20 ms; and scan range, 350–1800 *m*/*z*). This full-scan acquisition is subsequently followed by 15 MS/MS scans (resolution, 35,000; AGC target, 1 × 10^5^; and max IT, 100 ms). HCD collision energy was set to 32; precursor isolation window, 1.2 Da; and former target ion exclusion, 35 s.

#### 4.6.3. Data Analysis

Raw MS data were processed using MaxQuant software (Version 1.6.6) integrated with the Andromeda database search algorithm. The database used for retrieval is Swissprot. Human, 20210312 Proteome Database. The detailed search parameters were configured as below: TMT-based quantification mode was enabled. Variable modifications included oxidation of methionine (M), protein N-terminal acetylation, and deamidation of asparagine/glutamine (N/Q). Cysteine (C) carbamidomethylation (fixed modification) was set, and enzymatic digestion was specified as trypsin/P. Mass tolerance: 20 ppm (first search), 4.5 ppm (main search, MS1), and 20 ppm (MS2). Search results were filtered to 1% FDR (peptide/protein levels). Subsequently, decoy hits, contaminants, and proteins identified only by modified sites were eliminated, and valid identifications were subjected to quantitative. Identified proteins were annotated against the GO/KEGG databases via BLASTp alignment with an E-value threshold of ≤1 × 10^−5^ (Blast, 2.8.1 http://blast.ncbi.nlm.nih.gov/Blast.cgi, accessed on 1 March 2024). Differentially expressed proteins (DEPs) were screened out by calculating the fold change (FC). Those satisfying FC > 1.2 or FC < 0.83 and *p* < 0.05 (corresponding to |log_2_FC| > 0.263) were defined as DEPs. A volcano plot was generated to visualize the variation trends of DEPs, and functional enrichment analyses (R(clusterProfiler), 3.10.1) were performed to annotate DEPs to GO terms and KEGG pathways.

### 4.7. Proteasome Activity Determination

Proteasome chymotrypsin-like activity was assessed using the Proteasome-Glo™ Chymotrypsin-Like Cell-Based Assay Kit (Promega, G8660, Madison, WI, USA). Concisely, 100 µL proteasome-Glo™ reagent was mixed with 100 μL (50 μg) of hUCMSCs-EVs and hPPSCs-EVs samples, respectively. The mixture was transferred to a 96-well plate, mixed at 700 rpm for 2 min at room temperature, and then incubated for 10 min. After incubation, a microplate reader (Thermo Scientific, Varioskan LUX, Waltham, MA, USA) was used to quantify proteasome activity via the chemiluminescence module at a temperature of 37 °C.

### 4.8. The Number of Adherent Cells Assay

Endothelial cells were quantified using ImageJ software (National Institute of Health, version 1.53c, Bethesda, MD, USA). Each experimental group included 3 biological replicates (3 independent wells). For each biological replicate, all non-overlapping 200× fields of view with sufficient cells (ranging from 1 to 3 depending on the well’s cell density) were captured. The cell counts from all available fields per well were averaged to generate a single representative value for that biological replicate. Perform image processing and cell counting according to the following parameters (particle parameters, size 350-Infinity pixels^2^; circularity, 0.00–1.00).

### 4.9. Statistical Analysis

All experiments were performed with three independent biological replicates (n = 3). Data in the present work were presented as mean ± SD. Two independent groups were compared via an independent samples t-test, whereas multi-group (≥3) comparisons used one-way ANOVA. *p* < 0.05 indicated statistical significance. Statistical analyses and graphing were done via GraphPad Prism (v8.0.1).

## 5. Conclusions

hPPSCs-EVs contain chymotrypsin-like active proteasomes that degrade HIF-1α and ubiquitinated proteins, offering a novel strategy for ischemic–hypoxic disease treatment. Specifically, hPPSCs-EVs deliver exogenous 20S proteasomes to regulate HIF-1α and mitigate hypoxic damage. Abnormal HIF-1α accumulation is closely related to autoimmune diseases, poor cancer prognosis, and hypoxic diseases [25,70,71]. Therefore, this study demonstrates for the first time that the EV-20S proteasome participates in cell regulation by degrading key signaling molecules. This mechanism may enable cells to adapt to hypoxic conditions, providing a novel therapeutic approach for alleviating the tissue damage associated with these disorders. To further substantiate the conclusion regarding the role of EV-associated proteasomes, future studies will consider employing proteasome inhibition in donor cells or directly in isolated EVs. In addition, future experiments will focus on the purification and characterization of proteasomes within EVs, as well as on conducting in vivo functional studies. The identification of endothelial cell receptors that interact with extracellular vesicles, the tracking of intracellular trafficking of extracellular vesicles, and the functional differences between extracellular vesicles from different sources will also be key priorities in our future studies.

## 6. Patents

The patent related to this paper is for Junjun Xu (Application Number: 202511366607.6).

## Data Availability

The original contributions presented in this study are included in the article. Further inquiries can be directed to the corresponding author Junjun Xu.

## Abbreviations

The following abbreviations are used in this manuscript:

HIF-1α	Hypoxia-inducible Factor 1-alpha
EV	Extracellular Vesicle
hUVECs	Human Umbilical Vein Endothelial Cells
hPPSCs	Human Placenta-derived Perivascular Stem Cells
hUCMSCs	Human Umbilical Cord Mesenchymal Stem Cells
PHD	Prolyl Hydroxylase
VHL	Von Hippel–Lindau
UPS	Ubiquitin–proteasome System
MVBs	Multivesicular Bodies
ILVs	Intraluminal Vesicles
